# Impact of blood glucose on cognitive function in insulin resistance: novel insights from ambulatory assessment

**DOI:** 10.1038/s41387-024-00331-0

**Published:** 2024-09-11

**Authors:** Judith R. Gruber, Alea Ruf, Elena D. Süß, Sewin Tariverdian, Kira F. Ahrens, Carmen Schiweck, Ulrich Ebner-Priemer, Sharmili Edwin Thanarajah, Andreas Reif, Silke Matura

**Affiliations:** 1 Goethe University Frankfurt, University Hospital, Department of Psychiatry, Psychosomatic Medicine and Psychotherapy, Frankfurt am Main, Germany; 2 Mental mHealth Lab, Institute of Sports and Sports Science, Karlsruhe Institute of Technology (KIT), Karlsruhe, Germany; 3 Fraunhofer Institute for Translational Medicine and Pharmacology ITMP, Frankfurt am Main, Germany; 4 Max Planck Institute for Metabolism Research, Cologne, Germany

**Keywords:** Diabetes complications, Risk factors

## Abstract

**Background/objectives:**

Insulin resistance (IR)-related disorders and cognitive impairment lead to reduced quality of life and cause a significant strain on individuals and the public health system. Thus, we investigated the effects of insulin resistance (IR), and blood glucose fluctuations on cognitive function under laboratory and free-living conditions, using ecological momentary assessment (EMA).

**Subjects/methods:**

Baseline assessments included neuropsychological tests and blood analysis. Individuals were classified as either insulin-sensitive (<2) or insulin-resistant (≥2), based on their Homeostatic Model Assessment (HOMA-IR) values. Continuous glucose monitoring (CGM) using a percutaneous sensor was performed for 1 week. Using multiple linear regression, we examined the effects of HOMA-IR and CGM metrics on cognitive domains. Working memory (WM) performance, which was assessed using EMA, 4 times a day for 3 consecutive days, was matched to short-term pre-task CGM metrics. Multilevel analysis was used to map the within-day associations of HOMA-IR, short-term CGM metrics, and WM.

**Results:**

Analyses included 110 individuals (mean age 48.7 ± 14.3 years, 59% female, *n* = 53 insulin-resistant). IR was associated with lower global cognitive function (*b* = −0.267, *P* = 0.027), and WM (*b* = −0.316; *P* = 0.029), but not with executive function (*b* = −0.216; *P* = 0.154) during baseline. EMA showed that higher HOMA-IR was associated with lower within-day WM performance (β = −0.20, 95% CI −0.40 to −0.00). CGM metrics were not associated with cognitive performance.

**Conclusions:**

The results confirm the association between IR and decrements in global cognitive functioning and WM, while no effects of CGM metrics were observed, making IR a crucial time point for intervention. Targeting underlying mechanisms (e.g., inflammation) in addition to glycemia could be promising to minimize adverse cognitive effects. Registered under https://drks.de/register/de identifier no. DRKS00022774.

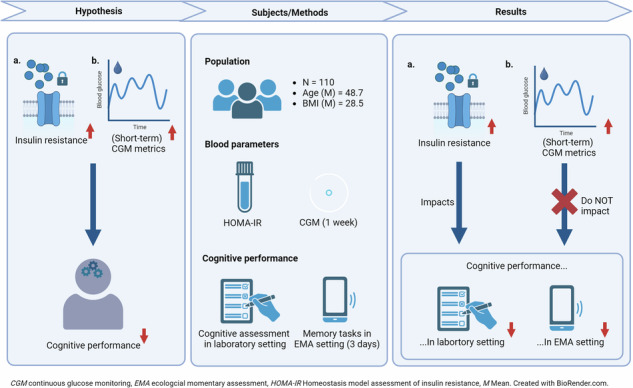

## Introduction

In 2021, type 2 diabetes affected over 529 million people globally, regardless of origin, age and sex [1]. A significant concern associated with type 2 diabetes is the increased risk of dementia and cognitive dysfunction [2]. Cognitive decrements are already evident in the early stages of insulin resistance (IR) and prediabetes [2, 3]. These impairments and IR-related somatic disorders diminish life quality and expectancy, placing a significant strain on the public health system and society as a whole [4]. Therefore, it is essential to understand underlying mechanistic changes, to enable preventative interventions.

IR is characterized by reduced sensitivity of the body’s cells to insulin, which diminishes the efficacy of the hormone and thereby leads to an overproduction of insulin (i.e., hyperinsulinemia) [5]. Insulin not only regulates peripheral blood glucose uptake but also passes the blood-brain barrier [6]. In the brain, central insulin signaling influences whole-body glucose metabolism and brain functions such as neurotransmission, synaptic plasticity, and neuroprotection [6–8]. Brain insulin resistance, like systemic IR, is characterized by a diminished response of brain cells to insulin [7] and reduced receptor uptake across the blood-brain barrier [6]. This has been observed in people with obesity and type 2 diabetes, normal ageing, and dementia [6]. Insulin receptors are widely distributed throughout the brain, suggesting that impaired insulin signaling may impact cognitive performance through multiple mechanisms. For one, obesity-related insulin resistance impacts several brain regions, including the hippocampus and prefrontal cortex, which are important for memory function [6, 9]. Further, proposed IR-related pathways that affect memory function are vascular brain injury, impaired brain glucose metabolism, inflammation, oxidative stress, and higher tau biomarkers [6, 7, 10]. Despite the established link between IR and cognitive function, specific mechanisms remain unclear.

Several studies have demonstrated a link between peripheral hyperinsulinemia and IR to reduced cognitive performance independent of type 2 diabetes [11–15]. For instance, a relationship between IR and diminished cognitive function was found in a middle-aged sample [12], and in prediabetic individuals aged 44–82 years [15]. In line with these findings, elevated fasting insulin levels have been linked to cognitive decline, particularly categorical verbal fluency [14]. Additionally to those cross-sectional studies, several studies provide longitudinal evidence on the relationship between IR and cognitive functions [16–18]. In a Korean cohort study of individuals over 65 years with normal cognition, increased IR was linked to diminished global cognitive function at the 6-year follow-up [16]. Moreover, Neergaard et al. [17] identified an association between IR and reduced verbal fluency in cognitively healthy postmenopausal women. However, heterogeneous definitions of IR, impaired glucose tolerance, and cognitive impairment in these studies hamper identifying underlying mechanisms.

In addition to the association between IR and reduced cognitive performance, previous studies indicate a link between glycemic control and cognitive function, both in individuals without diabetes [19, 20] and individuals with type 2 diabetes [21–24]. In individuals without diabetes, higher average glucose values were linked to an increased risk of dementia [19] and greater glucose fluctuations within higher frequency cycles showed adverse impacts on cognitive performance [20]. Notably, several studies examined glycemic control in individuals with type 2 diabetes by calculating continuous glucose monitoring (CGM) metrics [21–24]. For instance, glucose excursions were linked to reduced global cognitive function in older patients (78 ± 6.7 years) with type 2 diabetes [21], and in a Chinese sample of older (>65 years) individuals with type 2 diabetes [22]. Similarly, in a Japanese study with older (>70 years) patients with type 2 diabetes, acute hyperglycemia was associated with lower performance in executive function (EF), while better glucose control was associated with better executive and working memory (WM) function [23]. However, these studies address a cross-sectional relationship, do not exploit the analysis possibilities of CGM metrics (e.g., within-day fluctuations), and do not answer the question if glycemic control already affects cognitive performance in IR.

Technological advancements, such as CGM, have made data collection easier, facilitating exploratory research approaches beyond traditional laboratory settings. CGM, unlike HbA_1c_ measurements, offers a detailed view of glucose fluctuations over hours (short-term) or days, tracks excursions and daily profiles, and offers direct information for treatment and lifestyle modifications [25]. Recent studies used CGM and ecological momentary assessment (EMA) in type 1 diabetes research [26, 27] and also in exploring the relationship between CGM metrics and diabetes distress in type 2 diabetes [28]. EMA, an established method to collect data in individuals’ daily lives, offers advantages such as free-living data collection and analysis, and the reduction of retrospective biases [29]. Thus, EMA is crucial to expand the understanding of cognitive performance in everyday life and allows researchers to map variations in cognitive performance and fluctuations in acute glucose measures, as well as identify predictors and influences [27].

Using this novel approach, this study adds to the current knowledge on cognitive function in individuals with IR and the impact of glucose fluctuations in this context. This is accomplished by using the baseline assessment as well as the CGM metrics of an entire week. Second, this study aimed to elucidate the relationship between IR, glucose levels, and within-day WM performance in individuals using real-time data collection. For this, WM performance was assessed using EMA and combined with short-term CGM metrics (1 h before WM tasks). We postulated the following hypotheses for both the laboratory and free-living conditions: 1) IR is associated with lower cognitive performance, and 2) (Short-term) glucose fluctuations lead to diminished cognitive performance.

## Material/subjects and methods

Data from the present study were collected within the *m*PRIME study, a prospective, observational study of the H2020 project PRIME (grant no. 847879). Study recruitment took place between March 2021 and March 2023 at the University Hospital Frankfurt, Germany.

### Study population

All participants in the *m*PRIME study provided written informed consent. The study protocol and procedures were approved by the local ethics committee (reference number: 20-767) and registered with the German Clinical Trials Register (DRKS00022774). The study was conducted in accordance with the Code of Ethics of the World Medical Association (Declaration of Helsinki, 1975).

Individuals were included in the study according to the following criteria: age above 18 years, no existing type 1 diabetes or gestational diabetes, no intake of antidiabetic medications, insulin or glucocorticoids, no weight-reducing medications or diets, no severe neurological diseases, no diagnosis of lifetime bipolar I disorder, schizophrenia, organic mental disorders, or substance abuse, and no current pregnancy or breastfeeding. Individuals were screened for eligibility via a telephone interview and categorized as either insulin-resistant (*n* = 53) or insulin-sensitive (*n* = 57) using the diagnostic criteria Homeostasis Model Assessment of Insulin Resistance (HOMA-IR) [30] at baseline. IR was defined using a cut-off value of ≥2 [31]. Further, the Mini-Mental State Examination (MMSE) with a cut-off score of <25 [32] and the Multiple Choice Vocabulary Intelligence Test with a cut-off score of <75 [33] were administered to exclude individuals with existing cognitive impairment.

### Study design

**Fig. 1:**
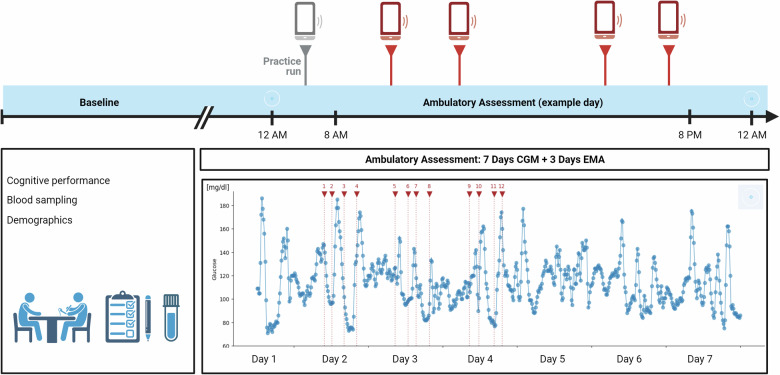
Design of the *m*PRIME study. Individuals completed a baseline assessment during which sociodemographic data were collected, blood samples were taken, and neuropsychological tests were carried out. Individuals were then familiarized with the ecological momentary assessment and fitted with a CGM sensor. The CGM sensor was worn continuously for 1 week (sensor icon). On 3 of the days (2 weekdays and 1 weekend day), individuals were randomly prompted to complete a working memory task (red triangles) at least four times per day between 8 a.m. and 10 p.m., while the CGM sensor was worn continuously (12 a.m. to 12 a.m.; see example day). CGM continuous glucose monitoring, EMA ecological momentary assessment. Created with BioRender.com.

Individuals completed the baseline assessment and a 1-week ambulatory assessment. At baseline, sociodemographic information and blood samples were collected, BMI and waist circumference were measured, and an extensive neuropsychological test battery was performed (see Supplementary Table S1 for details). Further, individuals were introduced to smartphone-based EMA (WM task), and a CGM sensor was applied. EMA was then completed for 3 consecutive days, including 2 weekdays and 1 weekend day (Thursday to Saturday or Sunday to Tuesday). On the first day, a 1-time button was included to allow the users to start a practice run of the WM task. At least four times a day, individuals were semi-randomly prompted (random prompts within a certain time interval) to perform a WM task between 8 am and 10 pm, with at least 1 h between tasks. After each WM task, individuals were asked to rate their concentration. The CGM sensor was worn continuously for 1 week. Glucose values were not visible for the individuals. An illustration of the design of the *m*PRIME study is provided in Fig. 1.

### Blood samples

All blood samples were obtained after an overnight fasting period of at least 8 h. Blood was collected (S-Monovette Serum-Gel CAT, 4.7 ml; S-Monovette Kalium EDTA 1.8 ml; Sarstedt) by venipuncture between 06.00 and 10.00 a.m., before testing procedures. Blood samples were sent to the central laboratory of the hospital to obtain the HOMA-IR (fasting insulin [mU/l] × fasting glucose [mg/dl])/405) [30].

### Measures

#### CGM metrics

CGM was conducted using the Freestyle Libre Pro IQ sensor (Abbott Diabetes Care, Alameda, United States of America). The sensor automatically records interstitial blood glucose levels every 15 min. Only individuals for whom glucose monitoring data was available for at least 3 days were included in the analysis. All measures were derived from raw data files. The R-package *iglu* [34] was used to compute the following CGM metrics for baseline analysis: mean sensor glucose (SG), standard deviation (SD), mean amplitude of glucose excursion (MAGE), and coefficient of variation (CV). Within the EMA setting, we calculated short-term CGM metrics for mean SG, and CV for 1-h windows before WM tasks.

#### Cognitive performance at baseline

Cognitive domains assessed at baseline comprised global function, WM, and EF. Global function was assessed using the following neuropsychological tests: Rey Complex Figure test [35], phonemic (letter ‘S’) and semantic (category “animals”) verbal fluency [36], Trail Making-Test A (TMT A) and B (TMT B) [37], Digit Span Forward and Backward [38], Block Span Forward and Backward [38] and Letter-Number-Span [39]. Further, the WM domain was calculated using the Digit Span Forward and Backward, Block Span Forward and Backward, and the Letter-Number-Span tests. EF included phonemic and semantic verbal fluency and TMT B. All raw scores were *z*-standardized for better interpretation. The z-scores of the TMT-A and B were inverted so that higher scores represent better performance. A global cognitive z-score was calculated by averaging the individual z-scores of each test. WM z-score and EF z-score were calculated accordingly.

#### WM performance during EMA

The WM task based on tasks developed by Riediger et al. [40] was implemented into the movisensXS app (version 1.5.11., movisens GmbH). The WM task was a numerical memory-updating task, which required individuals to perform simple additions and subtractions.

The WM task consisted of two parts: 1) three runs with three digits and 2) three runs with four digits (total duration ~2.5 min). An illustration of the sequence of the WM task is provided in the Supplements (Supplemental Figure S1). Individuals received performance feedback after each part. To assess momentary WM performance, the percentage of correct responses across all six trials was calculated. Concentration (Were your thoughts on what you were doing or on something else?) was self-reported on a visual analogue scale from 0 (on something else) to 100 (I was focused) after each WM task.

### Data pre-processing for EMA analysis

Each WM assessment was paired with the short-term CGM metrics mean SG and CV in the hour before the respective WM task. Individuals were excluded from analysis if they completed less than a pre-defined 30% of the prompts [41] or if no CGM metrics were captured during the EMA assessment. In the second step, the first trial (practice run, *n* = 101-time intervals) and outliers (|*z*-scores| > 3; *n* = 14-time intervals) with performance scores below 25% in WM performance—assuming guessing of responses—were excluded from analysis [40].

The Level-1 predictors’ mean SG, CV, and concentration were centered on the person-mean to generate unbiased estimates of the within-person effect. The trial number (first trial = 0) was included to control potential learning effects. Level-2 predictors were centered on fixed grand-means, to increase interpretability of the model intercept [42]. Age was centered around 49 years, and education years around 16. Sex was coded as 0 (male) and 1 (female) and group as 0 (insulin-sensitive) and 1 (insulin-resistant). HOMA-IR as well as CV and mean SG were transformed with the natural logarithm before analyses due to a right-skewed distribution. Data pre-processing and analyses were performed using SPSS (IBM, version 27.0.0.0), R (version 4.3.1 (2023-06-16 ucrt)), and Rstudio (version 2023.06.1.524).

### Data analysis

Descriptive statistics are reported as means ± SD or median [Interquartile range] for continuous variables and as proportions for categorical variables. Group differences were assessed using Mann-Whitney U test for continuous variables, and Fisher’s exact test for categorical variables. For baseline analysis, three multiple linear regression models with cognitive domains as dependent variables were performed to test the hypotheses in the laboratory setting. All models included the CGM-derived predictor CV and group (0 = insulin-sensitive, 1 = insulin-resistant) and were adjusted for age, sex, and education years. The analyses were repeated with mean SG instead of CV as a CGM-derived predictor. CV and mean SG were transformed with the natural logarithm before analyses due to a right-skewed distribution. The assumptions for multiple linear regressions (Variance inflation factor below 2, Breusch-Pagan test, Durbin-Watson test, and Shapiro-Wilk test) were tested and verified. The included sample size allowed 95% statistical power to detect moderate-to-large effect sizes in multiple linear regressions with five predictors (alpha = 0.05). A significance level of *P* < 0.05 was used for all tests.

Spearman correlation was run to determine the correlation between baseline WM performance and WM assessed during EMA, to assure adequacy of the test. Intraclass correlation (ICC) was calculated for the WM task to determine the degree to which WM performance varied between individuals or within individuals (between time points). A multilevel regression was used to map the nested data structure (time points [Level 1] nested within individuals [Level 2]) and to test the hypotheses under real-world conditions. To account for the restricted range of the dependent variable WM performance between 0 and 100, a beta regression was estimated using the R-package *brms* [37], which supports Bayesian multilevel modeling. To ensure all values of the dependent variable fall within the supported range of beta regressions (>0 to <1), WM performance was divided by 101 (e.g., 100%/101 = 0.99). Two models were calculated to assess the short-term effects of 1) high glucose values (mean SG), and 2) glucose variability (CV) on within-day cognitive performance. All models include a random intercept (we expect people to have different average WM performance) and a random slope for the short-term CGM metrics to examine differences between individuals. Additionally, the trial number of the WM task, concentration, age, sex, and education years were included as covariates. Model parameters were estimated based on 10.000 iterations. A sensitivity analysis for the multilevel models, which included only individuals with ≥7 trials, was carried out. *Brms* (version 2.19.0) and *rstan* (version 2.26.22) were used to perform the analyses. Credible intervals (95% CI) that do not include 0 indicate a significant effect.

## Results

Of the 124 individuals who started the study, 6 dropped out due to the substantial time commitment required for participation. One individual was excluded from the analysis due to a MMSE score <25. Another individual was excluded because of an existing untreated type 2 diabetes without meeting the criteria for IR. Further, CGM metrics could only be calculated for 110 participants, as some sensors did not record data (*n* = 5) or only for a short period of time (<3 days) (*n* = 1). Thus, the final sample included in baseline analysis was 110 participants.

### Baseline characteristics

Baseline characteristics and CGM metrics for the total sample and separated by insulin-sensitive and insulin-resistant groups are presented in Table 1. The total sample included in the multiple linear regression analyses consists of 110 participants (female 59%) with an average age of 48.7 $\pm$ 14.3 years. Insulin-sensitive (*n* = 57) and insulin-resistant (*n* = 53) groups differed significantly in age, years of education, BMI, C-reactive protein (CRP), and CGM metrics (*P* < 0.05). The insulin-resistant group was older than the insulin-sensitive group and showed higher values in metabolic and CGM metrics. There was no difference in the sex distribution between the two samples (*P* = 0.563).

**Table 1:** Baseline sociodemographic characteristics and CGM metrics of the total sample (*N* = 110) and for the insulin-sensitive and insulin-resistant groups separately.

	All (*N* = 110)	Insulin-sensitive (*n* = 57)	Insulin-resistant (*n* = 53)	*P* value^a,b^
Sex, *n*				
Female	65 (59.1)	32 (56.1)	33 (62.3)	0.563
Male	45 (40.9)	25 (43.9)	20 (37.7)	
Age, years	48.7 ± 14.3	45.4 ± 15.2	52.3 ± 12.4	0.009
Education, years	15.6 ± 2.9	16.3 ± 2.4	14.8 ± 3.2	0.019
BMI, kg/m^2^	28.5 ± 6.1	24.7 ± 2.7	32.7 ± 6.1	0.000
BMI ≤ 24.9	39 (35.5)	34 (59.6)	5 (9.4)	0.000
BMI 25 to 29.9	36 (32.7)	20 (35.1)	16 (30.2)	
BMI ≥ 30	35 (31.8)	3 (5.3)	32 (60.4)	
Waist circumference, cm	97.1 ± 15.9	87.7 ± 11.2	107.3 ± 13.9	0.000
CRP, mg/dl	0.1 [0.05–0.24]	0.05 [0.04–0.11]	0.19 [0.09–0.43]	0.000
HbA_1c_, mmol/mol	37 ± 4.4	34 ± 3.3	39 ± 5.5	0.000
HOMA-IR	2.7 ± 2.3	1.2 ± 0.4	4.3 ± 2.4	0.000
Untreated type 2 diabetes, *n*	4 (3.6)	0 (0)	4 (7.5)	0.051
CGM metrics				
MAGE	39.8 ± 13.1	35.9 ± 8.6	44.0 ± 15.7	0.004
Mean SG, mg/dl	95.3 ± 11.5	91.6 ± 7.3	99.2 ± 13.8	0.001
Glucose variability (CV), %	16.5 ± 3.8	15.8 ± 3.2	17.2 ± 4.3	0.038
SD, mg/dl	15.7 ± 4.5	14.4 ± 2.7	17.2 ± 5.5	0.004

*BMI* body mass index*, CGM* continuous glucose monitoring, *CRP* C-reactive protein, *CV* coefficient of variation (glucose variability), *EMA* ecological ambulatory assessment, *HOMA-IR* homeostasis model assessment of insulin resistance, *MAGE* mean amplitude of glucose excursions, *SD* standard deviation, *SG* sensor glucose.

Data are means ± SD, median [Interquartile range], or *n* (%).

^a^Fisher exact test for categorical variables.

^b^Mann-Whitney U test for continuous variables.

### Laboratory setting: relationship between IR, CGM metrics, and cognitive function

**Fig. 2:**
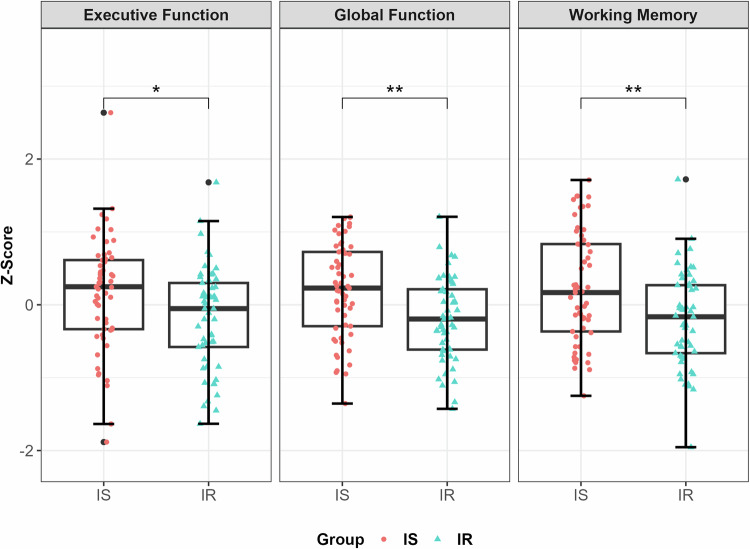
Boxplots with the z-scores for the three cognitive domains separated by groups. The boxplots illustrate the distribution of z-scores for the three cognitive domains—executive function, global function, and working memory—among IS and IR groups. The executive function domain assessed phonemic and sematic verbal fluency and Trail Making-Test B. Global function included Rey Complex Figure test, phonemic and semantic verbal fluency, Trail Making-Test A and B, Digit Span Forward and Backward, Block Span Forward and Backward, and Letter-Number-Span. Working memory included Digit Span Forward and Backward, Block Span Forward and Backward, and Letter-Number-Span. The red dots represent the IS group, while the turquoise rectangles represent the IR group. Higher z-scores indicate better cognitive performance. IS insulin-sensitive, IR insulin-resistant. * *P* < 0.05; ** *P* < 0.01.

In a first step, group differences in cognitive function were visually assessed. A boxplot with the z-scores for the three cognitive domains’ global function, WM, and EF separated by insulin-sensitive and insulin-resistant group is provided in Fig. 2. Detailed results of each of the neuropsychological tests and cognitive domains are included in the Supplements (Supplementary Table S1).

Further, the effects of IR and glucose variability, measured by CV, on the cognitive domains global function, WM, and EF were tested in three multiple linear regression models (Table 2). Being insulin-resistant was associated with reduced global function (*b* = −0.267, *P* = 0.027) and reduced WM performance (*b* = −0.316; *P* = 0.029), but not with EF (*b* = −0.216; *P* = 0.154). All models showed that glucose variability (CV) was no significant predictor of cognitive performance. Age was a significant predictor of global function and WM. Similar results were obtained for mean SG, as a measure for acute blood glucose, and are shown in the Supplements (Supplementary Table S2).

**Table 2:** Multiple linear regression models with cognitive domains as dependent variables.

Effect	Estimate	SE	95% CI		*P* value
			LL	UL	
Model 1: Global function^a^					
Intercept	0.225	0.733	−1.229	1.679	0.760
Glucose variability^b^	0.093	0.259	−0.420	0.606	0.720
Age	−0.015	0.004	−0.024	−0.007	0.000
Sex	0.115	0.114	−0.112	0.342	0.319
Education	0.022	0.020	−0.018	0.061	0.285
Group	–0.267	0.119	−0.503	−0.031	0.027
Model 2: Working memory^a^					
Intercept	0.205	0.880	−1.541	1.951	0.816
Glucose variability^b^	0.147	0.311	−0.469	0.764	0.636
Age	–0.016	0.005	−0.026	−0.006	0.002
Sex	0.104	0.138	−0.169	0.376	0.452
Education	0.017	0.024	−0.031	0.065	0.478
Group	−0.316	0.143	−0.600	−0.032	0.029
Model 3: Executive function^a^					
Intercept	0.455	0.925	−1.380	2.290	0.624
Glucose variability^b^	−0.160	0.327	−0.808	0.488	0.626
Age	−0.009	0.005	−0.020	0.001	0.072
Sex	0.168	0.144	−0.119	0.454	0.248
Education	0.029	0.025	−0.022	0.079	0.260
Group	−0.216	0.150	−0.514	0.082	0.154

*CI* confidence interval, *LL* lower limit, *UL* upper limit, *SE* standard error.

*N* = 110 (Insulin-sensitive = 57, Insulin-resistant = 53). Sex (0 = male, 1 = female), Group (0 = insulin-sensitive, 1 = insulin-resistant).

^a^Scores were z-standardized for better interpretation.

^b^Glucose variability was measured by the coefficient of variation (CV) which was log-transformed to correct right-skewed data.

### Free-living conditions: association between short-term CGM metrics, and WM performance in IR assessed with EMA

To explore the temporal relationship of short-term CGM metrics and WM performance in IR under free-living conditions, two multilevel analyses were performed using data from EMA. For this analysis, seven individuals were excluded due to low compliance (less than 30% of WM tasks completed). The final EMA dataset included 103 individuals (insulin-sensitive *n* = 54; insulin-resistant *n* = 49) and 1.052 time-points. Overall, the WM performance measured under free-living conditions was 86.43% (±10.87 between individuals) and significantly correlated with WM measured at baseline (*r*_*S*_ = 0.65, *P* < 0.000), suggesting an adequate reflection of WM performance. Descriptive statistics of the multilevel regression variables and the result of the ICC are included in the Supplements (Supplementary Table S3).

Results of the two multilevel models assessing the effect of mean SG and glucose variability on WM performance are shown in Table 3. Estimates of the multilevel models are presented as log-odds. The inverse logit function (e.g., R function plogis) was used to convert the log odds in proportion of correct responses (WM performance). In model 1, the intercept is 0.853 (plogis[1.76]), equaling a WM performance of 85.3%, when all predictors and covariates are equal to 0, or in the case of centered variables equal to the mean. For HOMA-IR (*β* = −0.20, 95% CI −0.40 to −0.00) the proportion of correct answers within the WM task is 0.826 (plogis[1.76–0.20]; 82.6%). Thus, a 1% increase in HOMA-IR above the grand-mean with all other covariates remaining at 0 or equal to the mean, is significantly associated with a 2.7% decrease in WM performance (0.853−0.826 = 0.027 = 2.7%). Mean SG has no significant fixed effect on WM performance, suggesting that between-person differences in acute blood glucose did not influence WM performance. However, there are fixed effects (between-person) of trial number, concentration, and age. This suggests that (1) there was a learning effect after repeated use of the test, (2) self-perceived concentration had an effect on test performance, and (3) age had an effect on WM performance. Mean SG, trial number, and self-reported concentration had no random effects. This suggests that there a no between time-point effects on WM performance. In Model 2, comparable results were found with glucose variability (CV) as a predictor. However, HOMA-IR had no significant fixed effect here.

**Table 3:** Within-day level: model estimates of the multilevel models with Level-1 predictors mean SG and glucose variability (CV) and dependent variable working memory.

Effect	Estimate	SE	95% CI	
			LL	UL
Model 1 − mean SG				
Fixed effects				
Intercept	1.76	0.12	1.53	1.98
Mean SG^a^	0.00	0.00	−0.00	0.00
Trial Number	0.03	0.01	0.01	0.04
Concentration^b^	0.01	0.00	0.01	0.02
HOMA-IR^c^	−0.20	0.10	−0.40	−0.00
Age	−0.02	0.01	−0.03	−0.01
Sex	0.15	0.15	−0.14	0.45
Education	0.04	0.03	−0.01	0.10
Random effects				
SD (Intercept)	0.72	0.07	0.60	0.86
SD (Mean SG^a^)	0.00	0.00	0.00	0.01
SD (Trial Number)	0.02	0.01	0.00	0.05
SD (Concentration^b^)	0.01	0.00	0.00	0.01
Model 2 − Glucose variability (CV)				
Fixed effects				
Intercept	1.73	0.13	1.48	1.98
Glucose variability^d^	−0.00	0.00	−0.01	0.01
Trial Number	0.03	0.01	0.01	0.04
Concentration^b^	0.01	0.00	0.01	0.02
HOMA-IR^c^	−0.19	0.10	−0.39	0.02
Age	−0.02	0.01	−0.03	−0.01
Sex	0.17	0.15	−0.13	0.47
Education	0.05	0.03	− 0.01	0.10
Random effects				
SD (Intercept)	0.75	0.08	0.62	0.92
SD (Glucose variability^d^)	0.01	0.01	0.00	0.03
SD (Trial Number)	0.02	0.01	0.00	0.04
SD (Concentration^b^)	0.01	0.00	0.00	0.01

Estimates of the two multilevel models are presented as log-odds. The inverse logit function (e.g., R function plogis) was used to convert the log-odds to proportion of correct responses (WM performance) for data interpretation.

*CV* coefficient of variation, *CI* confidence interval, *HOMA-IR* homeostasis model assessment of insulin resistance, *LL* lower limit, *SE* standard error, *SG* sensor glucose, *UL* upper limit. Sex (0 = male, 1 = female).

*N* = 103.

^a^Mean SG was log-transformed to correct right-skewed data.

^b^Self-reported item.

^c^HOMA-IR was log transformed to correct right-skewed data.

^d^Glucose variability is measured by the coefficient of variation (CV) which was log-transformed to correct right-skewed data.

Sensitivity analysis was performed to confirm the results. The results are presented in the Supplements (Supplementary Table S4).

## Discussion

Given the limited understanding of IR-related mechanisms on cognitive functions, this study investigates the relationship between IR and cognitive performance in both laboratory and free-living conditions by integrating CGM and EMA sampling. Our main findings are: 1) Compared to insulin-sensitive individuals, insulin-resistant individuals show decrements in global cognitive function and WM, but not EF, when tested in the laboratory. An increase in HOMA-IR was associated with reduced WM performance during everyday life. 2) (Short-term) CGM metrics do not correlate with cognitive domains or within-day WM performance. The results suggest that in our sample increased insulin production (i.e. hyperinsulinemia) was sufficient to prevent large glucose variability. Small variations in glucose variability could explain the lack of a direct effect of blood glucose on cognition. Suggesting, that the overall diminished performance in global function and WM in our insulin-resistant group was most likely a result of IR-related mechanistic pathways (i.e. brain insulin resistance, inflammation, oxidative stress) caused by glycemic changes. Age emerged as a significant covariate across all models.

Our findings confirm that cognitive function decrements develop early in impaired glucose metabolism, e.g. insulin resistance, independent of type 2 diabetes diagnosis [3]. Baseline cognitive performance differences between insulin-resistant and insulin-sensitive individuals align with previous findings [11–18]. However, our results did not replicate findings for diminished EF among individuals with IR [14, 17, 18]. Methodological differences in capturing EF, such as our use of a composite z-standardized score, may explain this discrepancy.

Further, we investigated the relationship between CGM metrics and baseline cognitive performance. Unlike studies on individuals with type 2 diabetes [21–24], we found no significant association between glucose variability (CV) or mean SG and cognitive performance. There are several possible explanations. First, most of these studies targeted older individuals diagnosed with type 2 diabetes, whereas our sample comprised middle-aged individuals. Therefore, different mechanisms might impact cognitive function. Second, most studies employed a single measure for cognitive performance, while we used a comprehensive neuropsychological test battery. Lastly, the low glucose variability and stable controlled blood glucose levels in our sample, even when IR was present, might explain the lack of an association between CGM metrics and cognitive performance. Although surprising at first glance, the results confirm other studies investigating glycemia in IR or prediabetes that showed that effects of IR on cognition are independent of glycemia [15, 43]. Willmann et al. [15] suggested that cognitive decline may be caused by IR-induced mechanistic changes, especially brain insulin resistance, rather than by increased blood glucose [6, 15]. Further, potential underlying mechanistic changes that have been discussed concerning IR include inflammation caused by oxidative stress, with chronic inflammation leading to tissue changes in the periphery and brain [6].

Consistent with our baseline analysis, the EMA analysis showed a negative association between HOMA-IR and WM performance but not with short-term CGM metrics. This suggests that IR has a small yet significant impact on everyday cognitive function. Previous studies assessed IR and cognitive function in laboratory settings without being able to draw implications for free-living conditions. Our study highlights the value of EMA as a complementary tool [28] to established neuropsychological test batteries by providing a more comprehensive picture. However, we observed ceiling effects in our WM task, questioning the sensitivity to capture small cognitive changes. Given the subtle nature of cognitive changes in IR [2], it is important to use sensitive tools in EMA settings capable of detecting minor cognitive changes [44] and high-frequency usage, to avoid ceiling effects [27].

### Strength and limitations

This study’s strength lies in its comprehensive approach that integrates objective and self-reported measures in laboratory and real-life settings.

However, the observational study design precludes establishing causal relationships. Second, while HOMA-IR is an established surrogate marker for IR, there is no official consensus on cut-off values and considerable variability in reported thresholds. The cut-off value of 2 used in our study may not detect effects that manifest at more advanced stages of IR. Further, HOMA-IR alone may not capture subtle postprandial alterations in glucose and insulin production that occur early in the diabetes progression. Third, the chosen observational window for short-term CGM metrics (the hour before each WM task) does not capture the full impact of glucose fluctuations that expand beyond 1 h, potentially limiting variability in the data and missing effects. While previous studies adopted CV and mean SG as informative acute glucose metrics, other measures might be superior in capturing short-term differences and providing a more comprehensive picture. Fourth, significant group differences in metabolic, glycemic, and sociodemographic factors, especially BMI and diabetes status, may confound results, potentially introducing bias and affecting interpretation. Although differences in age, sex, and education were taken into account in the analyses, we did not adjust for BMI, and thus cannot rule out its influence. The results need therefore be confirmed in matched groups.

## Conclusions

Our results show that in our middle-aged sample, IR rather than (short-term) CGM metrics is associated with cognitive function decrements. The cognitive impacts of IR might be small but seem to have an early onset and are part of the diverse impacts of IR (e.g., brain insulin resistance and inflammatory pathways). Thus, IR appears to be a crucial time point for intervention, at which targeting lifestyle such as nutrition and physical exercise to restore insulin sensitivity and minimize glycemia could serve as a promising approach. Further research is needed to better understand the mechanisms underlying insulin-related cognitive decrements, which is important to inform early prevention measures and help minimize the impact on quality of life.

## Supplementary information

Supplemental Material

## Data Availability

The datasets generated during the current study are available in the Zenodo repository, 10.5281/zenodo.11235373.
