# Supplementary material for: Impact of blood glucose on cognitive function in insulin resistance: novel insights from ambulatory assessment
Source: Nutr Diabetes. 2024 Sep 11;14:74. doi: 10.1038/s41387-024-00331-0 (PMC11390747; doi:10.1038/s41387-024-00331-0)
Supplement: Supplementary file 1 — Supplemental Material [file 41387_2024_331_MOESM1_ESM.pdf]

Supplementary Materials

To: Impact of Blood Glucose on Cognitive Function in Insulin Resistance: Novel Insights from Ambulatory Assessment

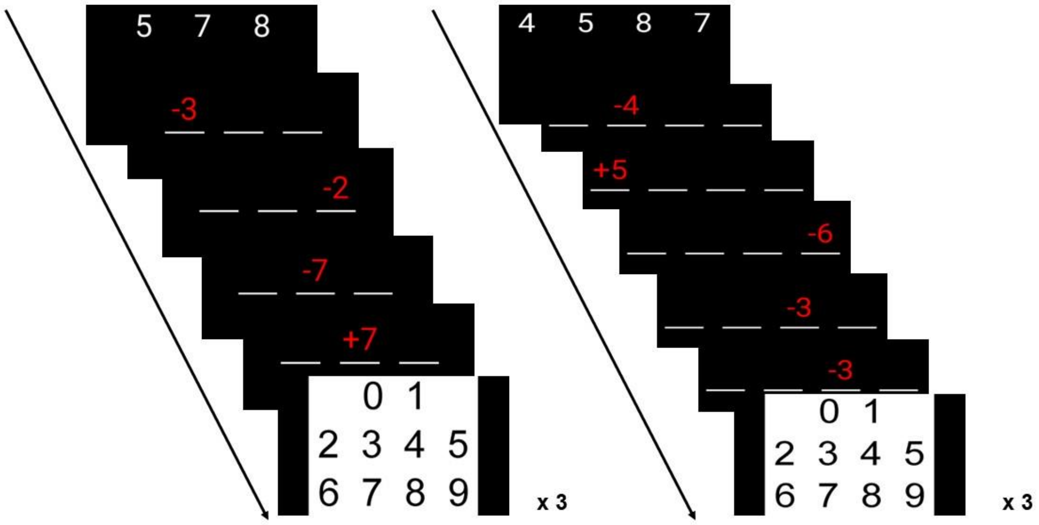

Supplemental Figure S1 – Sequence of working memory tasks during EMA. The WM task consisted of 2 parts: 1.) 3 runs with 3 digits and 2.) 3 runs with 4 digits. After memorizing the sequence (first row), individuals were presented with alternating addition or subtraction tasks below a given digit position. After 4 (5) operations, they selected the resulting digit sequence from a range of 0 to 9 (last row), taking care that the digits did not fall below 0 or above 9. This procedure was performed 3 times for each sequence length.

Supplemental Table S1 – Cognitive assessment at baseline of the total study population and for the insulin-sensitive and insulin-resistant groups separately

	**All (N = 110)**	**Insulin-sensitive (n = 57)**	**Insulin-resistant (n = 53)**	***P* value**[a]
MMSE, n	30 [29-30]	30 [29-30]	29 [29-30]	0.022
Rey-Complex Figure Test	125 [100-152]	137.5 [100-156]	119 [97-136]	0.069
Digit Span, n				
Forward	8 [7-10]	8 [7-10]	8 [7-10]	0.405
Backward	7 [6-8]	7 [6-9]	6 [6-8]	0.055
Block Span, n				
Forward	9 [8-10]	10 [8-11]	9 [8-10]	0.034
Backward	9 [8-10]	10 [8-11]	8 [7-10]	0.001
Letter-Number-Span, n	21 [19-23]	21 [20-24]	20 [18-22]	0.011
Verbal Fluency, n				
Letter “S”,	21.5 [18-26]	23 [19-29]	20 [16-24]	0.040
Animals	38 [33-42]	38 [33-43]	37 [33-42]	0.353
Trail Making-Test, s				
A	26.85 [21.81-34.14]	25.53 [19.33-31.36]	30.19 [24.8-38.05]	0.007
B	59.61 [47.54-83]	54 [43.61-65]	73 [53.08-88.58]	0.002
Cognitive domains				
Global cognition[b,c]	0.034 [-0.471-0.402]	0.230 [–0.293-0.725]	–0.195 [–0.615-0.213]	0.001
Working memory[b,d]	–0.053 [–0.573-0.509]	0.168 [–0.366-0.834]	–0.165 [–0.664-0.269]	0.008
Executive function[b,e]	0.103 [–0.469-0.426]	0.248 [–0.334-0.614]	–0.052 [–0.579-0.299]	0.013

Data are median [interquartile range]. [a]Mann-Whitney U test. [b]All raw scores were z-standardized for better interpretation. Z-Scores of Trail Making Test were inverted. [c]Global cognition: Average z-score of Rey Complex Figure Test, Verbal Fluency, Trail Making-Test, Digit Span, Block Span, Letter-Number-Span. [d]Working Memory: Average z-score of Digit Span, Block Span, Letter-Number-Span, [e]Executive function: Average z-score of Verbal Fluency, Trail Making-Test B

Supplemental Table S2 - Multiple linear regression models with cognitive domains as dependent variables

Effect	Estimate	SE	95 % CI		*P value*
			LL	UL	
Model 1: Global function[a]					
Intercept	0.744	2.647	–4.504	5.993	0.779
mean SG[b]	–0.065	0.575	–1.205	1.075	0.910
Age	–0.015	0.004	–0.023	–0.007	0.000
Sex	0.110	0.119	–0.127	0.347	0.359
Education	0.022	0.020	–0.018	0.062	0.276
Group	–0.257	0.122	–0.500	–0.015	0.038
Model 2: Working memory[a]					
Intercept	2.003	3.176	–4.295	8.302	0.529
mean SG[b]	–0.317	0.690	–1.685	1.051	0.647
Age	–0.015	0.005	–0.025	–0.005	0.004
Sex	0.084	0.143	–0.200	0.368	0.559
Education	0.017	0.024	–0.031	0.065	0.491
Group	–0.289	0.147	–0.580	0.002	0.052
Model 3: Executive function[a]					
Intercept	–3.763	3.320	–10.346	2.820	0.260
mean SG[b]	0.842	0.721	–0.588	2.271	0.246
Age	–0.012	0.005	–0.022	–0.001	0.027
Sex	0.218	0.150	–0.079	0.516	0.148
Education	0.032	0.025	–0.0181	0.082	0.208
Group	–0.273	0.153	–0.577	0.031	0.078

N = 110 (Insulin-sensitive = 57, Insulin-resistant = 53). *CV* coefficient of variation, *CI* confidence interval, *LL* lower limit, *UL* upper limit, *SE* standard error. Sex (0 = male, 1 = female); Group (0 = Insulin-sensitive, 1 = Insulin-resistant). [a]Scores were z-standardized for better interpretation. [b]Mean SG was log-transformed to correct right-skewed data.

Supplemental Table S3 - Descriptive statistics of multilevel model parameters

	**All (N = 103)**	**Insulin-sensitive (n = 54)**	**Insulin-resistant (n = 49)**	***P* value[a]**
Time points, n	1.052	560	492	
Trials, n	10.21 ± 2.62	10.37 ± 2.67	10.04 ± 2.59	0.381
Level-1 predictors				
mean SG, mg/dl	94.74	91.63	98.28	0.000
within individuals	± 14.76	± 13.09	± 16.48	
between individuals	± 11.05	± 7.04	± 13.47	
Glucose variability (CV), %	6.72	6.71	6.72	0.813
within individuals	± 6.05	± 6.08	± 5.96	
between individuals	± 3.56	± 3.30	± 3.75	
working memory performance, %	86.43	88.95	83.57	0.000
within individuals	± 13.29	± 11.61	± 14.48	
between individuals	± 10.87	± 8.14	± 12.55	

Data are means ± SD or *n*. *CV* coefficient of variation, *SG* sensor glucose. [a]Mann-Whitney U test.

Results of the ICC:

The ICC was 0.40 for WM performance during EMA (adjudicating an amount of 60% within individual variance).

Supplemental Table S4 - Sensitivity analysis on within-day level: Model estimates of the multilevel model with Level-1 predictor mean SG and glucose variability (CV) and dependent variable working memory

Effect	Estimate	SE	95% CI	
			LL	UL
Model 1 – mean SG				
Fixed effects				
Intercept	1.83	0.12	1.58	2.07
Mean SG[a]	0.00	0.00	–0.00	0.00
Trial Number	0.02	0.01	0.01	0.04
Concentration[b]	0.01	0.00	0.01	0.02
HOMA-IR[c]	–0.21	0.10	–0.41	–0.00
Age	–0.01	0.01	–0.02	–0.00
Sex	0.11	0.16	–0.20	0.43
Education	0.03	0.03	–0.03	0.08
Random effects				
SD (Intercept)	0.70	0.07	0.57	0.84
SD (Mean SG[a])	0.00	0.00	0.00	0.01
SD (Trial Number)	0.02	0.01	0.00	0.04
SD (Concentration[b])	0.01	0.00	0.00	0.01
Model 2 – Glucose variability (CV)				
Fixed effects				
Intercept	1.82	0.13	1.55	2.07
Glucose variability[d]	–0.00	0.00	–0.01	0.01
Trial Number	0.02	0.01	0.00	0.04
Concentration[b]	0.01	0.00	0.01	0.02
HOMA-IR[c]	–0.21	0.11	–0.42	0.00
Age	–0.01	0.01	–0.02	–0.00
Sex	0.12	0.16	–0.19	0.44
Education	0.03	0.03	–0.03	0.08
Random effects				
SD (intercept)	0.72	0.08	0.58	0.88
SD (Glucose variability[d])	0.01	0.01	0.00	0.02
SD (Trial Number)	0.02	0.01	0.00	0.04
SD (Concentration[b])	0.01	0.00	0.00	0.01

N = 90. *CGM* continuous glucose monitoring parameter (Model 1. mean SG; Model 2. CV), *CV* coefficient of variation, *CI* confidence interval, *SE* standard error, *SG* sensor glucose, *LL* lower limit, *UL* upper limit, *HOMA-IR* homeostasis model assessment of insulin resistance, Sex (0 = male, 1 = female). [a]mean SG was log-transformed to correct right-skewed data. [b]Self-reported item. [c]HOMA-IR was log transformed to correct right-skewed data. [d]Glucose variability is measured by the coefficient of variation (CV) which was log-transformed to correct right-skewed data. Estimates of the two multilevel models are presented as log-odds. The inverse logit function (e.g., R function plogis) was used to convert the log-odds to proportion of correct responses (WM performance) for data interpretation.
